# A Comparison of Rosuvastatin Monotherapy and Rosuvastatin Plus Ezetimibe Combination Therapy in Patients With Type 2 Diabetes: A Meta-Analysis of Randomized Controlled Trials

**DOI:** 10.7759/cureus.61526

**Published:** 2024-06-02

**Authors:** Samuel K Dadzie, Godfrey Tabowei, Mandeep Kaur, Saeed Ahmed, Aayushi Thakur, Khaldoun Khreis, Monika Bai, Adil Amin

**Affiliations:** 1 Internal Medicine, Piedmont Athens Regional Medical Center, Athens, USA; 2 Internal Medicine, Texas Tech University Health Sciences Center, Odessa, USA; 3 Hospital Medicine, HCA Florida Capital Hospital, Tallahassee, USA; 4 Cardiology, Mohtarma Benazir Bhutto Shaheed Medical College, New Mirpur City, PAK; 5 Medicine, Tbilisi State Medical University, Tbilisi, GEO; 6 Pediatrics, University of Pécs Medical School, Pécs, HUN; 7 Obstetrics and Gynaecology, Sheikh Zayed Hospital for Women, Larkana, PAK; 8 Cardiology, PNS (Pakistan Navy Ship) Shifa Hospital, Karachi, PAK

**Keywords:** systematic review and meta analysis, type 2 diabetes, monotherapy, ezetimibe, rosuvastatin

## Abstract

Type 2 diabetes mellitus is a metabolic condition where vascular inflammation and oxidative stress contribute to disease progression and associated complications. Although statins are recommended for managing dyslipidemia in diabetes, additional therapies are often required to achieve target lipid levels. This meta-analysis aimed to evaluate the efficacy of rosuvastatin monotherapy versus combination therapy with ezetimibe in patients with type 2 diabetes. A systematic literature search was conducted across multiple databases until April 2024, identifying six randomized controlled trials meeting the inclusion criteria. The meta-analysis revealed that the rosuvastatin plus ezetimibe combination resulted in significantly greater reductions in total cholesterol (mean difference, or MD: 19.49; 95% CI: 13.99 to 24.99), triglycerides (MD: 13.44; 95% CI: 2.04 to 24.85), and low-density lipoprotein cholesterol (MD: -17.68; 95% CI: 12.85 to 22.51) compared to rosuvastatin monotherapy. Conversely, rosuvastatin monotherapy achieved a greater reduction in HbA1c levels (MD: -0.11; 95% CI: -0.17 to -0.04). Subgroup analysis demonstrated that using the same dose of rosuvastatin in both groups led to more significant improvements in lipid parameters with lower heterogeneity. The findings suggest that the rosuvastatin-ezetimibe combination may be a more effective lipid-lowering strategy for patients with type 2 diabetes, though larger studies are needed to assess long-term safety and optimal dosing. Additionally, while rosuvastatin monotherapy provided modest HbA1c reductions, the clinical relevance remains uncertain, and potential risks with high-dose statins should be considered.

## Introduction and background

Type 2 diabetes mellitus is a long-term metabolic condition where changes in the immune system play a role in the disease's development and the advancement of its associated complications, such as cardiovascular disease [1]. Individuals with diabetes can experience various forms of dyslipidemia [1]. A significant risk factor for cardiovascular disease in patients with metabolic syndrome and type 2 diabetes is a characteristic combination known as the atherogenic triad: hypertriglyceridemia, reduced high-density lipoprotein cholesterol (HDL-C), and increased levels of small dense low-density lipoprotein (LDL) [2]. The Framingham Study found that the prevalence of elevated LDL-C was similar between diabetic patients and their non-diabetic counterparts, with rates of 9% and 15% in diabetic men and women, respectively, compared to 11% and 16% in non-diabetics [3] . Similarly, the United Kingdom Prospective Diabetes Study (UKPDS) reported no significant difference in total cholesterol levels between diabetics and non-diabetics, although LDL-C levels were comparable in men but higher in women with type 2 diabetes compared to those without the condition [4]. The development of diabetic dyslipidemia is complex, with insulin resistance and an increased flux of free fatty acids to the liver playing a central role [2]. This promotes the typical dyslipidemia triad of high plasma triglyceride levels, low plasma HDL-C, and an increased concentration of small dense LDL particles. The elevated free fatty acids enhance hepatic triglyceride production, leading to a greater secretion of apoB and very low density lipoprotein (VLDL). Triglycerides transported by VLDL are exchanged for cholesteryl esters carried by HDL through the action of cholesteryl ester transfer protein (CETP). This process results in an increase in both atherogenic, cholesterol-rich VLDL remnant particles and triglyceride-rich, cholesterol-depleted HDL particles [2].

Most clinical guidelines recommend statin use for patients with diabetes and dyslipidemia. If the target levels for LDL-C are not reached with statin treatment monotherapy, the addition of ezetimibe is advised [5]. Rosuvastatin is among the potent HMG-CoA reductase inhibitors available, capable of reducing LDL-C by up to 55% [6]. It also has additional positive effects on the cholesterol profile, including raising HDL-C by about 6%, lowering triglycerides by 15% or more, and reducing the cholesterol content in atherosclerotic plaques [6]. When compared to other statins, rosuvastatin offers several benefits, one of which is its hydrophilicity, linked to a very low incidence of rhabdomyolysis and myopathy. It can also be taken at any time of day due to its extended duration of effect [7]. As the only medication in its class, ezetimibe lowers LDL-C by around 15%-20% by blocking NPC1L1, which can reduce cholesterol absorption by up to 67% [8]. Apart from its anti-inflammatory properties, ezetimibe plus statin medication reduces high-sensitivity C-reactive protein by around 10% more compared to statin therapy alone [9]. A combination of rosuvastatin and ezetimibe is now available commercially in doses of 10/10 mg, 20/10 mg, and 40/10 mg [10]. While statins lower lipid levels by decreasing endogenous cholesterol production in the liver, the body compensates by increasing cholesterol absorption, which can reduce the efficacy of statins. Adding ezetimibe helps by inhibiting cholesterol absorption, thereby enhancing the LDL-C-lowering effects of statins [10].

Historically, the combination of ezetimibe and statin has been studied with simvastatin, a moderate-intensity statin, showing positive results. More recently, the US Food and Drug Administration (FDA) and the European Medicines Agency (EMA) have approved the combination of rosuvastatin, a high-intensity statin, with ezetimibe [11]. This review aims to summarize the current evidence comparing the combination of rosuvastatin and ezetimibe to rosuvastatin alone in patients with type 2 diabetes. The findings could help develop more tailored therapeutic strategies and improve treatment efficacy for comorbidities associated with type 2 diabetes.

## Review

Methodology

The Preferred Reporting Items for Systematic Reviews and Meta-Analyses (PRISMA) guidelines served as the foundation for our meta-analysis.

Search Strategy

We found studies by exploring the following data repositories: PubMed, Web of Science, Embase and Cochrane Library, from the inception of databases to April 30, 2024. The following keywords and medical subject headings (MeSH) terms were utilized without any language restriction: “Rosuvastatin”, “Ezetimibe”, “ezetimibe-rosuvastatin drug combination” and “type 2 diabetes”. We also reviewed the reference lists of the identified studies to uncover potentially pertinent research. Two authors conducted the literature search independently, with a third reviewer resolving any discrepancies that arose.

Study Selection

The included studies were full-text peer-reviewed articles meeting the following criteria: (1) human studies, (2) studies examining the impacts of rosuvastatin/ezetimibe and rosuvastatin alone in individuals with type 2 diabetes, (3) studies employing a randomized controlled design, and (4) reporting at least one of the outcomes evaluated in this meta-analysis. We excluded animal studies and studies including patients other than type 2 diabetes patients. We also excluded observational studies, reviews, case reports, case series and editorials. Two author performed study selection in two phases. In the first phase, abstract and titles were screened. In the second phase, full text of all eligible articles was obtained, and based on the pre-defined inclusion and exclusion criteria, detailed assessment was done. Any disagreement in the process of study selection was resolved by the third investigator.

Data Extraction

Two authors conducted data extraction from the included studies. The extracted data encompassed the author's name, year of publication, dosage of drugs, sample size, duration of follow-up, patients' characteristics, and outcomes assessed in this meta-analysis. The outcomes evaluated in this meta-analysis included changes in total cholesterol, triglycerides, high-density lipoprotein cholesterol, low-density lipoprotein cholesterol, fasting plasma glucose (FPG), and hemoglobin A1c (HbA1c).

Quality Assessment

The evaluation of the quality of the studies included in the analysis was performed by applying the Cochrane tool designed for assessing the risk of bias in randomized controlled trials (RCTs). This tool systematically examines various aspects of trial methodology to gauge the likelihood of bias influencing the study outcomes.

Statistical Analysis

Statistical analyses were conducted utilizing RevMan software (Nordic Cochrane Centre, Cochrane Collaboration, Copenhagen). Typically, the mean and standard deviation of percentage changes as reported in the articles were utilized, and the pooled mean difference (MD) was presented alongside a 95% confidence interval (CI). A P-value less than 0.05 was considered significant. A random-effects meta-analysis was carried out using the limited maximum likelihood method. The heterogeneity among studies was assessed using tau-square and I-square statistics. An I-square value of 50% or more indicates significant heterogeneity among the study results. Forest plots were provided for each outcome assessed in this meta-analysis.

Results

**Figure 1:**
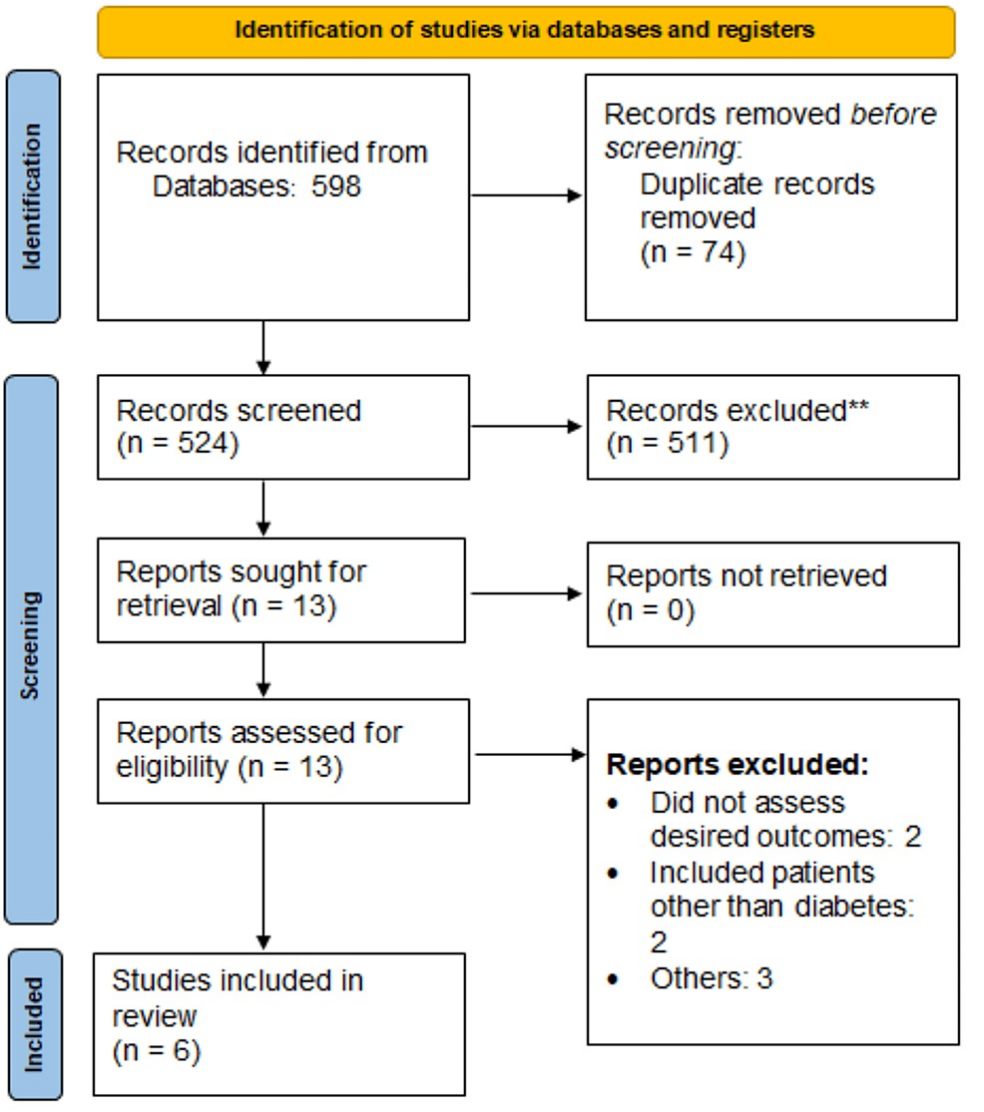
Preferred Reporting Items for Systematic Reviews and Meta-Analyses (PRISMA) flowchart of study selection **Records removed after title and abstract screening

**Figure 2:**
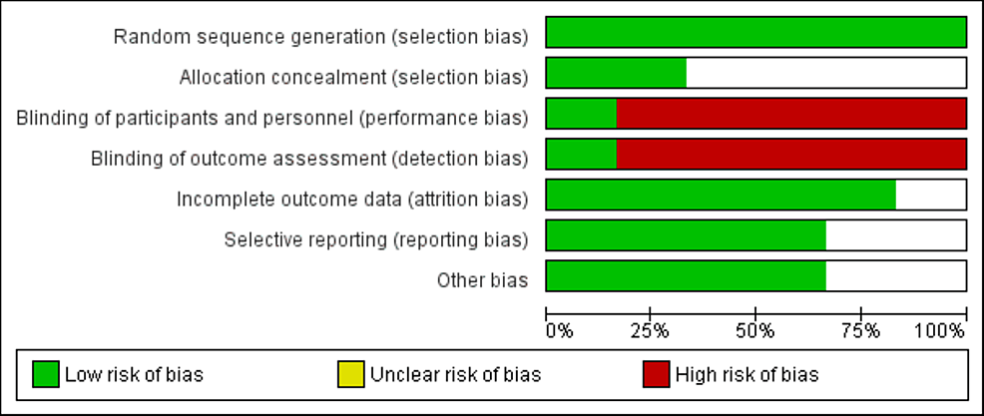
Risk of bias assessment of included studies

Out of the 598 articles initially identified, 524 underwent screening after the removal of 74 duplicate records. Among these, 13 studies proceeded to the next stage of detailed assessment. Ultimately, six RCTs were included in this meta-analysis. Figure 1 presents the PRISMA flowchart of study selection. Characteristics of included studies are shown in Table 1. Among the six included studies, three used the same dose of statin in both groups, while three studies used double the dose of statin in the monotherapy group compared to the combination therapy group. Follow-up duration in the included studies ranged from 6 to 12 weeks. Figure 2 shows the quality assessment of included studies assessed using the Cochrane tool for risk of bias assessment.

**Table 1:** Characteristics of included studies Age has been presented as means, and gender has been presented as N.

Author names and year	Groups	Population	Dose of rosuvastatin	Follow-up period	Mean age (years)	Males (N)
Han et al., 2024 [12]	Rosuvastatin	47	5 mg	12 Weeks	57.15	19
	Rosuvastatin/ezetimibe	45	5 mg		55.04	23
Hwang et al., 2019 [13]	Rosuvastatin	21	20 mg	6 Weeks	53	14
	Rosuvastatin/ezetimibe	21	5 mg		50.4	13
Ju et al., 2024 [14]	Rosuvastatin	74	5 mg	12 Weeks	56.9	33
	Rosuvastatin/ezetimibe	75	5 mg		53.6	35
Lee et al., 2020 [15]	Rosuvastatin	68	10 mg	8 Weeks	56.9	38
	Rosuvastatin/ezetimibe	68	5 mg		53.7	37
Torimoto et al., 2013 [16]	Rosuvastatin	36	5 mg	12 Weeks	63	16
	Rosuvastatin/ezetimibe	39	2.5 mg		66.3	24
Vaverkova et al., 2012 [17]	Rosuvastatin	82	10 mg	6 Weeks	64.5	45
	Rosuvastatin/ezetimibe	100	10 mg		63.1	58

Comparison of Two Treatments on the Basis of Change in Lipid Parameters

**Figure 3:**
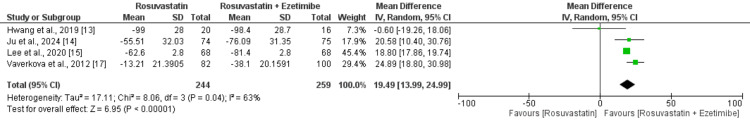
A comparison of change in total cholesterol levels between the two groups References [13-15,17]

**Figure 4:**
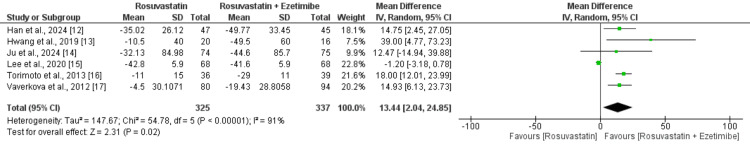
A comparison of change in triglycerides between the two groups References [12-17]

**Figure 5:**
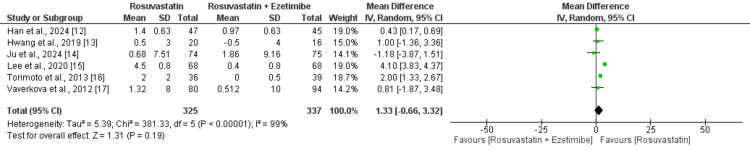
A comparison of change in high-density lipoprotein (HDL) levels between the two groups References [12-17]

**Figure 6:**
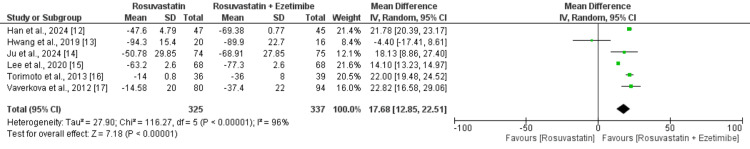
A comparison of change in low-density lipoprotein cholesterol (LDL-C) levels between the two groups References [12-17]

Based on four studies (I^2^ = 63%; P-value for heterogeneity = 0.04), the change in reduction in total cholesterol was greater in the rosuvastatin plus ezetimibe group compared to the rosuvastatin group (MD: 19.49; 95% CI: 13.99 to 24.99), as shown in Figure 3. The reduction in triglycerides from baseline was significantly higher in patients receiving rosuvastatin plus ezetimibe compared to patients in the rosuvastatin monotherapy group based on the pooled analysis of six RCTs (I^2^ =91%; P-value for heterogeneity <0.01) (MD: 13.44; 95% CI: 2.04 to 24.85), as shown in Figure 4. The HDL-C increase (I^2^ = 99%; P-value for heterogeneity <0.01) was not significantly different between the two groups based on the pooled analysis of six studies (MD: 1.33; 95% CI: -0.66 to 3.32), as shown in Figure 5. Lastly, reduction in levels of LDL-C was significantly higher in patients receiving rosuvastatin plus ezetimibe compared to patients in the rosuvastatin monotherapy group (MD: -17.68; 95% CI: 12.85 to 22.51) (I^2^ = 96%; P-value for heterogeneity <0.01), as shown in Figure 6.

Comparison of Two Treatments on the Basis of Change in Hb1AC and FPG

**Figure 7:**
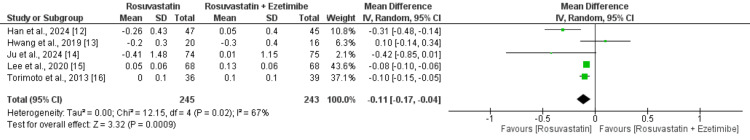
A comparison of change in HbA1C (%) between the two groups References [12-16]

**Figure 8:**
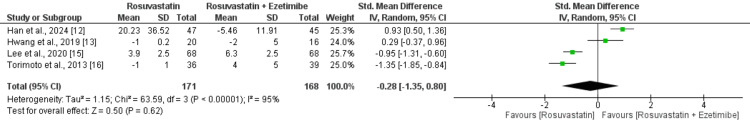
A comparison of change in fasting plasma glucose (FPG, mg/dl) levels between the two groups References [12-13,15-16]

Five studies compared the change in Hb1AC from baseline; the results are shown in Figure 7. A pooled analysis reported that the reduction in Hb1AC was significantly greater in patients receiving rosuvastatin monotherapy compared to the patients in the combination group (MD: -0.11; 95% CI: -0.17 to -0.04). On the contrary, the change in FPG from baseline was not significantly different between the two groups, as shown in Figure 8 (MD: -0.28; 95% CI: -1.35 to 0.80).

Subgroup Analysis

Table 2 presents the outcomes of subgroup analyses comparing the effects of the same dose of rosuvastatin in both groups and higher doses of rosuvastatin in monotherapy. For total cholesterol, triglycerides, HDL-C, and LDL-C, the same dose of rosuvastatin in both groups showed statistically significant improvements, with larger effect sizes and lower heterogeneity compared to higher doses of rosuvastatin in monotherapy. Regarding HbA1c and FPG, the same dose of rosuvastatin in both groups led to a significant reduction in HbA1c with low heterogeneity, and a significant increase in FPG with no heterogeneity. In contrast, higher doses of rosuvastatin in monotherapy resulted in a smaller reduction in HbA1c with moderate heterogeneity and a non-significant reduction in FPG with high heterogeneity.

**Table 2:** Subgroup analysis HDL-C: high-density lipoprotein cholesterol; LDL-C: low-density lipoprotein cholesterol; Hb1AC: glycated hemoglobin; FPG: fasting plasma glucose; MD: mean difference; CI: confidence interval

Outcomes	Subgroups	MD (95% CI)	I^2^
Total cholesterol	Same dose of rosuvastatin in both groups	23.75 (18.53 to 28.98)	0%
	Higher doses of rosuvastatin in monotherapy	11.43 (-7.02 to 29.89)	76%
Triglycerides	Same dose of rosuvastatin in both groups	14.72 (7.79 to 21.64)	0%
	Higher doses of rosuvastatin in monotherapy	13.31 (-4.50 to 31.12)	95%
HDL-C	Same dose of rosuvastatin in both groups	0.42 (0.16 to 0.67)	0%
	Higher doses of rosuvastatin in monotherapy	2.58 (0.74 to 4.42)	95%
LDL-C	Same dose of rosuvastatin in both groups	21.75 (20.41 to 23.09)	0%
	Higher doses of rosuvastatin in monotherapy	13.79 (6.21 to 21.36)	95%
Hb1AC	Same dose of rosuvastatin in both groups	-0.33 (-0.48 to -0.17)	10%
	Higher doses of rosuvastatin in monotherapy	-0.08 (-0.11 to -0.05)	32%
FPG	Same dose of rosuvastatin in both groups	0.93 (0.50 to 1.36)	0%
	Higher doses of rosuvastatin in monotherapy	-0.70 (-1.50 to 0.09)	87%

Discussion

This meta-analysis was conducted to determine the efficacy of rosuvastatin monotherapy and rosuvastatin and ezetimibe in patients with type 2 diabetes. The meta-analysis found that the reduction in lipid parameters including total cholesterol, triglycerides and LDL-C was significantly greater in patients receiving rosuvastatin and ezetimibe. In contrast, the reduction in Hb1AC was significantly greater in patients receiving rosuvastatin monotherapy compared to its counterparts. We performed a subgroup analysis to further explore how an increase in the dose of rosuvastatin in the monotherapy group affected the change in lipid parameters. Through subgroup analysis, we found that the reduction in total cholesterol and triglycerides was higher in patients receiving combination therapy, but the difference was statistically insignificant.

The statin and ezetimibe combination group experienced a higher LDL-C reduction compared to the double-dose statin monotherapy group, according to a prior meta-analysis that included 11 clinical studies [18]. A recent study has shown that statin/ezetimibe combination therapy provides additional cardioprotective effects compared with statin monotherapy, making it a viable choice to further lower LDL-C levels [11]. However, side effects associated with statin therapy are directly linked with the dose of the statin [19]. Furthermore, a recent meta-analysis demonstrated an inverse relationship between total and LDL-C levels and the risk of hemorrhagic stroke [20]. However, in the present meta-analysis, we analyzed higher doses of rosuvastatin separately, and except for LDL-C reduction, no significant differences were reported in other lipid parameters.

In recent times, evidence has emerged suggesting that a reduction in the size of LDL particles is linked with an increased coronary artery disease risk. Individuals who primarily have small, dense LDL particles (sdLDL-C) are known to have a three times greater incidence of heart disease compared to those with normal-sized LDL particles [21]. However, it has been shown that individuals with type 2 diabetes frequently have elevated levels of sdLDL-C, suggesting that preventing atherosclerosis requires both lowering LDL-C levels and growing the LDL particle size [22]. A previous study found that ezetimibe combination therapy and statin monotherapy had similar effects in reducing sdLDL-C levels [23]. This meta-analysis explicitly stated that irrespective of whether the dose of rosuvastatin is high or the same in the monotherapy group, the effect of the combination of ezetimibe and rosuvastatin on LDL-C reduction is positive. Ezetimibe acts by inhibiting the absorption of cholesterol in the intestines, while rosuvastatin reduces cholesterol synthesis in the liver. This dual mechanism of action results in a more pronounced reduction in LDL cholesterol levels than either agent alone. The synergistic effect may also involve modulation of cholesterol transport and metabolism at the cellular level, enhancing lipid clearance from the bloodstream [17].

In this study, the participants who received simultaneous treatment with ezetimibe experienced significant improvements in their total cholesterol, LDL-C, and triglyceride levels. The exact mechanism through which ezetimibe reduces triglyceride levels is not fully understood. However, it has been proposed that this medication may lower triglyceride levels by inhibiting cholesterol absorption, reducing chylomicron production, decreasing fatty acid absorption via FATP4, and suppressing apoB-48 production in the small intestine [24-25].

The meta-analysis revealed that rosuvastatin monotherapy was associated with a greater reduction in HbA1c levels compared to combination therapy (MD: -0.11; 95% CI: -0.17 to -0.04), which is consistent with previous studies demonstrating statins' potential glucose-lowering effects involving improved insulin sensitivity, enhanced insulin secretion, and decreased inflammation [26]. However, the modest HbA1c reduction may not be clinically significant for most patients, and the benefits should be weighed against risks like myopathy and new-onset diabetes with high-dose statins [27]. Regarding ezetimibe addition, some evidence suggests it may provide additive benefits by improving insulin sensitivity, reducing inflammation, and optimizing the lipid profile [27], but other studies found no significant difference in HbA1c between statin monotherapy and statin-ezetimibe combination [28]. While the primary aim of adding ezetimibe is more aggressive LDL lowering, any glycemic improvements would be a secondary effect, likely modest and dependent on factors like baseline control and treatment duration.

In the present meta-analysis, we were not able to compare the risk of adverse events between the two groups as only two out of six studies assessed the adverse events. However, in the future, we need further RCTs to compare the risk of treatment-related adverse events between the two groups at different doses to choose the best dosage of rosuvastatin in terms of efficacy and safety. The net advantage of the statin/ezetimibe combination has also received attention recently, coinciding with the publication of research demonstrating the therapeutic benefit of this combination. Reports, however, have not yet offered enough proof of the statin/ezetimibe combination's overall clinical advantage over high-intensity statins. Based on the safety and effectiveness of the two regimens with a similar LDL-C decrease, this should be estimated. It is important to note that the current meta-analysis compared two regimens that reduced LDL-C to remarkably similar levels. By doing this, we could generate useful and clinically relevant data.

Our meta-analysis has certain limitations too. Firstly, only a limited number of trials (six) were included, and there was a lack of consistency as most trials utilized different dosages of rosuvastatin, introducing potential variability in the results. This variability in dosages may have influenced the overall efficacy and comparability of the outcomes, complicating the interpretation of the data. Secondly, we were not able to comprehensively assess the safety profile of combination therapy, as only two of the six included studies evaluated adverse events. Thirdly, we were unable to perform subgroup analyses based on certain variables, including concomitant medication and comorbidities, which could have provided more nuanced insights into the therapy's effects across different patient populations. Future studies should aim to select and standardize the optimal dose of rosuvastatin in combination therapy to ensure more consistent and reliable results.

Our findings have significant clinical implications, suggesting that the combination of ezetimibe and rosuvastatin could offer superior lipid-lowering benefits compared to monotherapy. This could influence current clinical practices by encouraging the adoption of combination therapy for patients with hypercholesterolemia who do not achieve target lipid levels with monotherapy. Additionally, these results highlight the need for further research to refine dosing strategies and evaluate long-term safety, ultimately guiding more effective and individualized treatment plans.

## Conclusions

This meta-analysis demonstrated that the combination of rosuvastatin and ezetimibe provided superior reductions in total cholesterol, triglycerides, and LDL-C levels compared to rosuvastatin monotherapy in patients with type 2 diabetes. However, rosuvastatin monotherapy resulted in a greater reduction in HbA1c levels, though the clinical significance of this finding is questionable. The subgroup analysis revealed that using the same dose of rosuvastatin in both groups led to more significant improvements in lipid parameters with lower heterogeneity. Overall, the findings suggest that the rosuvastatin-ezetimibe combination may be a more effective lipid-lowering strategy. Nevertheless, larger studies are warranted to assess the long-term safety of treatment and determine the optimal dosing of this combination therapy to better guide clinical practice and ensure patient safety.

## References

[1] Ahmad E, Lim S, Lamptey R, Webb DR, Davies MJ (2022). Type 2 diabetes. Lancet.

[2] Mooradian AD (2009). Dyslipidemia in type 2 diabetes mellitus. Nat Clin Pract Endocrinol Metab.

[3] Kannel WB (1985). Lipids, diabetes, and coronary heart disease: insights from the Framingham Study. Am Heart J.

[4] U.K. Prospective Diabetes Study Group (1997). U.K. Prospective Diabetes Study 27. Plasma lipids and lipoproteins at diagnosis of NIDDM by age and sex. Diabetes Care.

[5] Stone NJ, Robinson JG, Lichtenstein AH (2014). 2013 ACC/AHA guideline on the treatment of blood cholesterol to reduce atherosclerotic cardiovascular risk in adults: a report of the American College of Cardiology/American Heart Association Task Force on Practice Guidelines. Circulation.

[6] Jones PH, Davidson MH, Stein EA (2003). Comparison of the efficacy and safety of rosuvastatin versus atorvastatin, simvastatin, and pravastatin across doses (STELLAR Trial). Am J Cardiol.

[7] McTaggart F (2003). Comparative pharmacology of rosuvastatin. Atheroscler Suppl.

[8] Sudhop T, Lütjohann D, Kodal A (2002). Inhibition of intestinal cholesterol absorption by ezetimibe in humans. Circulation.

[9] Jeu L, Cheng JW (2003). Pharmacology and therapeutics of ezetimibe (SCH 58235), a cholesterol-absorption inhibitor. Clin Ther.

[10] Chilbert MR, VanDuyn D, Salah S, Clark CM, Ma Q (2022). Combination therapy of ezetimibe and rosuvastatin for dyslipidemia: current insights. Drug Des Devel Ther.

[11] Cannon CP, Blazing MA, Giugliano RP (2015). Ezetimibe added to statin therapy after acute coronary syndromes. N Engl J Med.

[12] Han JH, Joung KH, Lee JC (2024). Comparative efficacy of rosuvastatin monotherapy and rosuvastatin/ezetimibe combination therapy on insulin sensitivity and vascular inflammatory response in patients with type 2 diabetes mellitus. Diabetes Metab J.

[13] Hwang YC, Jun JE, Jeong IK, Ahn KJ, Chung HY (2019). Comparison of the efficacy of rosuvastatin monotherapy 20 mg with rosuvastatin 5 mg and ezetimibe 10 mg combination therapy on lipid parameters in patients with type 2 diabetes mellitus. Diabetes Metab J.

[14] Ju SH, Lim JY, Song M (2024). Distinct effects of rosuvastatin and rosuvastatin/ezetimibe on senescence markers of CD8+ T cells in patients with type 2 diabetes mellitus: a randomized controlled trial. Front Endocrinol.

[15] Lee J, Hwang YC, Lee WJ (2020). Comparison of the efficacy and safety of rosuvastatin/ezetimibe combination therapy and rosuvastatin monotherapy on lipoprotein in patients with type 2 diabetes: multicenter randomized controlled study. Diabetes Ther.

[16] Torimoto K, Okada Y, Mori H (2013). Efficacy of combination of ezetimibe 10 mg and rosuvastatin 2.5 mg versus rosuvastatin 5 mg monotherapy for hypercholesterolemia in patients with type 2 diabetes. Lipids Health Dis.

[17] Vaverkova H, Farnier M, Averna M (2012). Lipid-altering efficacy of ezetimibe/simvastatin 10/20 mg compared to rosuvastatin 10 mg in high-risk patients with and without type 2 diabetes mellitus inadequately controlled despite prior statin monotherapy. Cardiovasc Ther.

[18] Yu M, Liang C, Kong Q, Wang Y, Li M (2020). Efficacy of combination therapy with ezetimibe and statins versus a double dose of statin monotherapy in participants with hypercholesterolemia: a meta-analysis of literature. Lipids Health Dis.

[19] Golomb BA, Evans MA (2008). Statin adverse effects: a review of the literature and evidence for a mitochondrial mechanism. Am J Cardiovasc Drugs.

[20] Wang X, Dong Y, Qi X, Huang C, Hou L (2013). Cholesterol levels and risk of hemorrhagic stroke: a systematic review and meta-analysis. Stroke.

[21] Austin MA, Breslow JL, Hennekens CH, Buring JE, Willett WC, Krauss RM (1988). Low-density lipoprotein subclass patterns and risk of myocardial infarction. JAMA.

[22] Feingold KR, Grunfeld C, Pang M, Doerrler W, Krauss RM (1992). LDL subclass phenotypes and triglyceride metabolism in non-insulin-dependent diabetes. Arterioscler Thromb.

[23] Winkler K, Jacob S, Müller-Schewe T, Hoffmann MM, Konrad T (2012). Ezetimibe alone and in combination lowers the concentration of small, dense low-density lipoproteins in type 2 diabetes mellitus. Atherosclerosis.

[24] Masuda D, Nakagawa-Toyama Y, Nakatani K (2009). Ezetimibe improves postprandial hyperlipidaemia in patients with type IIb hyperlipidaemia. Eur J Clin Invest.

[25] Sandoval JC, Nakagawa-Toyama Y, Masuda D (2010). Molecular mechanisms of ezetimibe-induced attenuation of postprandial hypertriglyceridemia. J Atheroscler Thromb.

[26] Alvarez-Jimenez L, Morales-Palomo F, Moreno-Cabañas A, Ortega JF, Mora-Rodríguez R (2023). Effects of statin therapy on glycemic control and insulin resistance: a systematic review and meta-analysis. Eur J Pharmacol.

[27] Dixit JV, Badgujar SY, Giri PA (2022). Reduction in HbA1c through lifestyle modification in newly diagnosed type 2 diabetes mellitus patient: a great feat. J Family Med Prim Care.

[28] Kater AL, Batista MC, Ferreira SR (2010). Synergistic effect of simvastatin and ezetimibe on lipid and pro-inflammatory profiles in pre-diabetic subjects. Diabetol Metab Syndr.

